# Improving the Thermostability of Serine Protease PB92 from *Bacillus alcalophilus* via Site-Directed Mutagenesis Based on Semi-Rational Design

**DOI:** 10.3390/foods12163081

**Published:** 2023-08-16

**Authors:** Huabiao Miao, Xia Xiang, Nanyu Han, Qian Wu, Zunxi Huang

**Affiliations:** 1 Engineering Research Center of Sustainable Development and Utilization of Biomass Energy, Ministry of Education, Kunming 650500, China; 2 School of Life Science, Yunnan Normal University, Kunming 650500, China

**Keywords:** protease, thermostability, B-factor, site-directed mutagenesis, weighted analysis

## Abstract

Proteases have been widely employed in many industrial processes. In this work, we aimed to improve the thermostability of the serine protease PB92 from *Bacillus alcalophilus* to meet the high-temperature requirements of biotechnological treatments. Eight mutation sites (N18, S97-S101, E110, and R143) were identified, and 21 mutants were constructed from B-factor comparison and multiple sequence alignment and expressed via *Bacillus subtilis*. Among them, fifteen mutants exhibited increased half-life (t_1/2_) values at 65 °C (1.13–31.61 times greater than that of the wild type). Based on the composite score of enzyme activity and thermostability, six complex mutants were implemented. The t_1/2_ values of these six complex mutants were 2.12–10.05 times greater than that of the wild type at 65 °C. In addition, structural analysis revealed that the increased thermal stability of complex mutants may be related to the formation of additional hydrophobic interactions due to increased hydrophobicity and the decreased flexibility of the structure. In brief, the thermal stability of the complex mutants N18L/R143L/S97A, N18L/R143L/S99L, and N18L/R143L/G100A was increased 4-fold, which reveals application potential in industry.

## 1. Introduction

Proteases, a class of enzymes responsible for protein peptide chain hydrolysis, hold significant industrial importance and find applications in various sectors, including tanning, silk production, feed processing, medicine, food production, and environmental protection. They have emerged as a dominant force in the global enzyme market, accounting for approximately 60% of the total market share [1,2]. Prior to this, serine protease from the *Bacillus alcalophilus* strain PB92 was isolated, and the gene encoding this enzyme has been cloned and overexpressed in other *Bacillus* expression systems [3]. The 269-residue serine protease PB92 is a member of the subtilase family of enzymes. The extracellular activity of protease PB92 exhibits good plasticity and the best pH value of extremely alkaline, which has attracted significant research interest from both academic and industrial circles, and it has been applied in modern industry [4,5]. Thermostability is a crucial characteristic desired for the successful commercial implementation of enzymes, as thermal denaturation often leads to enzyme inactivation [6]. The thermostability of protease PB92 cannot meet the requirements of industrial application scenarios in high-temperature washing, high-temperature textile processing, the high-temperature cooking of food, thermophilicity, and fermentation [1,2]. Consequently, the identification and development of proteases with robust thermostability have been a longstanding focus of research [7].

There are two main ways to obtain thermophilic proteases: one is to use biodiversity and genetic engineering techniques to obtain them from natural thermophiles, which can be isolated from some high-temperature environments, and then use genetic engineering technology to introduce the target genes of thermophilic bacteria into other hosts for overexpression [8,9,10]; another method is to use molecular directed evolution technology to produce a large number of mutants from a heterologous gene through in vitro mutation and recombination: after expression in the host bacteria, the thermophilase with a strong catalytic ability in a high-temperature environment is specifically screened out [8,11,12,13,14]. The main methods include (1) using homologous family sequence alignment: comparing room temperature proteins with their corresponding thermophilic homologous families, finding sites with different residues, and increasing the thermophilia of room temperature proteins via single mutation or combination mutation on these sites [15]; (2) using the interaction between molecular structures: on the basis of understanding the protein structure, various interactions in the protein (hydrogen bonds, salt bonds, hydrophobic interactions, disulfide bonds and embedding rate, etc.) are analyzed, one or more interaction forces for mutation are selected, and a mutation strategy to increase the thermal stability of the protein is ultimately proposed [16]; and (3) using support vector machines as the main methods for mathematical statistical calculation based on a large number of samples [17]. Directed evolution technology is highly targeted, and the number of residues that need to be analyzed and processed is small, which is more efficient for finding reasonable mutation sites [18]. The B-factor is closely related to the thermostability of proteins and is a fixed parameter in the crystal structure, which reflects the degree of uncertainty or ambiguity of each atom in a protein around its measured position, and the larger the B-factor value is, the more unstable the corresponding atom [19,20]. Mutants with significantly improved thermostability can be obtained by mutating amino acids with higher B-factors. For example, the previous modification of xylanase and lipase in our group used the difference in B-factors to determine the mutation site [21,22].

Previous results have shown that protease PB92 has extremely significant extracellular protease activity and a relatively low cost in the production process, but protease PB92 poses certain limitations for high-temperature applications [4,5]. Thermolysin is not only a highly efficient industrial enzyme for synthetic peptides, such as precursors of the artificial sweetener aspartame, but also a typical model enzyme for thermostability studies, with better temperature adaptability than serine proteases [23,24]. Thermolysins will be used as templates for multiple sequence alignments in our study. In the work described in this paper, aiming at the shortcomings of the weak heat tolerance of protease PB92, the key amino acid residues of protease PB92 were mutated using rationally designed methods to improve the thermal stability of protease PB92 and enable its industrial application scenarios.

## 2. Results

### 2.1. Prediction of Mutagenesis Sites Based on B-Factor Analysis

**Figure 1:**
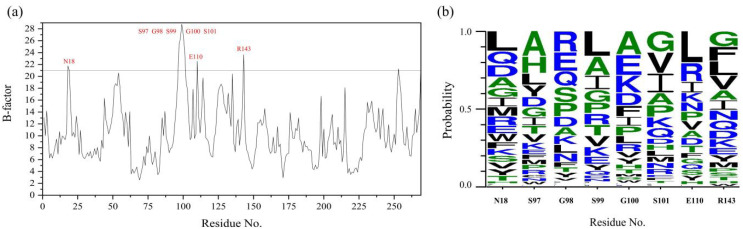
B-factor analysis and multisequence alignment of protease PB92: (**a**) B-factor values of C_α_ atoms for protease PB92; (**b**) 146 thermolysin sequences from different species of *Bacillus* via multiple sequence alignment.

In this study, the B-factor values specifically for the C_α_ atoms of PB92 were extracted using its corresponding crystal structure (Figure 1a). The B-factor values of N18, S97–S101, E110, R143, and S153 are over 21.0, exhibiting a high degree of flexibility. Considering that the S153 residue is located at the C-terminus of the protein structure, the secondary structure is mostly random coils and was rejected. Subsequently, the multiple sequence alignment used 146 thermolysin sequences obtained from *Bacillus* in the PDB database, and beneficial sequences from residues N18, S97–S101, E110, and R143 were thereby discovered for the amino acid residues with a frequency in the top 2 (Figure 1b) or more than 10% (Table S1). Taking into account the insights gained from the B-factor analysis and the multiple sequence alignment, the following putative sites were identified: N18L, N18Q, S97A, S97H, G98R, G98E, G98Q, S99L, S99A, G100E, G100A, G100K, S101G, S101I, S101V, E110L, E110R, R143L, R143V, R143G, and R143F. These residues show promise for improving the thermostability of PB92.

### 2.2. Construction and Characterization of Mutant Protease PB92

Twenty-one single-mutant forms of protease PB92 from B-factor analysis and multiple sequence alignment were prepared using site-directed mutagenesis. After the sequence accuracy of 21 single-mutant forms was verified, the recombinant plasmid was expressed using *B. subtilis* WB600. Mutants with extracellular protease activity, after overnight incubation on plates containing 1.0% skim milk powder, can form a proteolytic cycle (Figure S1). Most mutants can form hydrolysis circles over 20.0 mm. However, mutants N18Q, G98Q, G100K, S101I, E110R, and R143F either did not form proteolytic cycles or yielded smaller hydrolysis circles, which indicates that the extracellular protease activity of mutants was significantly reduced (Figure S1).

The extracellular protease activity of the wild type and 21 mutants was comparatively determined. The results showed that the relative extracellular protease activity values of mutants N18L, S97A, S97H, G98R, G98E, S99L, S99A, G100E, G100A, S101G, S101V, E110L, R143L, R143V, and R143G were 72.30%, 90.89%, 98.52%, 19.95%, 6.19%, 96.66%, 111.01%, 7.88%, 93.82%, 90.29%, 18.69%, 31.28%, 75.73%, 78.53%, and 98.24%, respectively (Table 1 and Figure S1). However, the extracellular protease activity of mutants N18Q, G98Q, G100K, S101I, E110R, and R143F was less than 5% of that of the wild type (Table 1 and Figure S2), so the following property studies will not be considered.

The optimal temperature for the wild type and 15 single mutants was determined over a temperature range (40–75 °C) at pH 10.5. The optimal temperature for wild-type protease PB92 was 55 °C. The optimal temperature values for mutants N18L, S99A, S99L, G100E, E110L, R143L, and R143G were all 60 °C, which was 5 °C higher than that of the wild type, and that of the mutant S101V even reached 65 °C. The mutants S97H, S97A, G98E, G98R, G100A, S101G, and R143V were maintained unchanged at 55 °C, as with the wild type (Table 1 and Figure S3).

**Figure 2:**
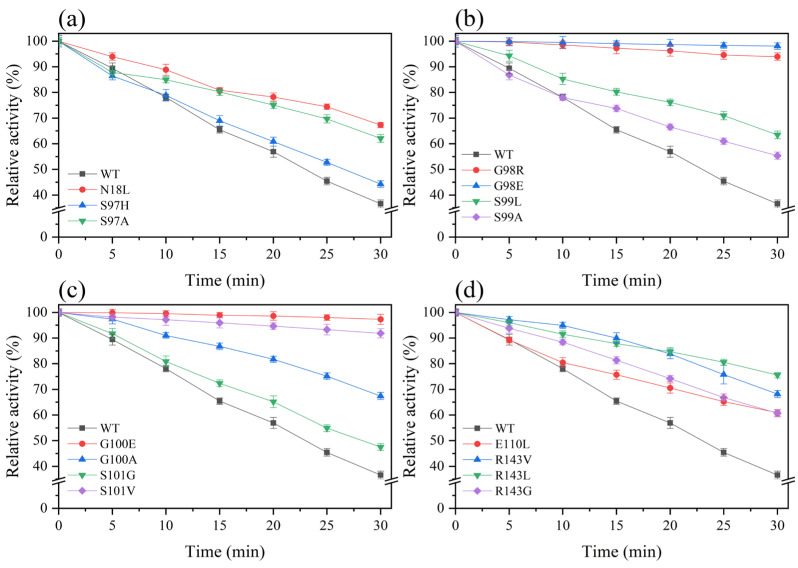
Temperature stability of the wild type and its mutants: (**a**) N18L, S97H, and S97A; (**b**) G98E, G98R, S99A, and S99L; (**c**) G100E, G100A, S101G, and S101V; (**d**) E110L, R143V, R143L, and R143G. WT represents an expression vector for the protease PB92. The enzymes were incubated for 30.0 min at 65 °C and demonstrated activity at pH 10.5 and 55 °C. The enzyme activity of protease without any heat treatment was established as the reference value of 100%.

The residual activities of the wild type and 15 different mutants were compared after different durations of treatment at 65 °C (Figure 2). The residual activity of the wild type decreased significantly with increasing heat treatment times. When the wild type was treated at 65 °C for 30 min, the residual activity was only 36.59% (Figure 2). The thermostability of mutants N18L and S97A was improved, and after 30.0 min, the residual activities were 67.29 and 62.09%, respectively (Figure 2a). The mutants S98R and S98E showed better thermostability, and the residual enzyme activity remained over 90.00% (Figure 2b). For the four mutations at the S99 and G100 sites, the residual activities were 57.45, 55.35, 97.31, and 67.42%, respectively (Figure 2b,c). In addition, mutant S101V also showed excellent thermostability, and the residual enzyme activity remained above 90.00% (Figure 2c). For the mutations of the E110 and R143 sites, the thermostability of the four mutants relative to the wild type was improved to varying degrees, and the residual activity of the mutants E110L, R143V, R143L, and R143G remained at 60.91, 68.20, 75.56, and 60.70%, respectively (Figure 2d). In summary, 15 single mutants (N18L, S97A, S97H, S98R, S98E, S99L, S99A, S101, E143, and R143) were treated at 65 °C for different durations. The residual activity of these mutants exhibited varying degrees of improvement compared to the wild-type protease PB92.

Next, the half-lives (t_1/2_) of different mutants at 65 °C were compared (Table 2). The t_1/2_ values of 15 mutants were all larger than that of the wild type (23.14 min), among which the mutant G98E had the best thermostability: its t_1/2_ was 731.39 min, which was more than 30 times that of the wild type. Following that, the t_1/2_ of the G100E mutant was 548.80 min, which was 23.72 times higher than that of the wild type, and the t_1/2_ values of mutants G98R and S101V were 230.71 min and 190.44 min, respectively, 8–10 times higher than that of the wild type. The t_1/2_ values of the remaining 11 mutants (N18L, S97A, S97H, S99L, S99A, G100A, S101G, E110L, R143V, R143L, and R143G) were 1.13–2.72 times higher than that of the wild type.

### 2.3. Identification of Composite Mutants

Through the study of the activity and thermostability of 15 mutants, it is not difficult to find significant inconsistency between the two relative to the amount of change in the wild type. Therefore, a composite score was introduced to identify complex mutants. The activity and thermostability (t_1/2_) of mutants relative to 1% changes in the wild type were defined as a score of 1, and the activity score and thermostability score of wild-type protease PB92 were defined as 100. We assumed that activity and thermostability were consistent with respect to importance to the protease PB92 and calculated the activity score, thermostability score, and composite score for each protease (Table 3). The results show that the composite score of the three mutants at the S101 and E110 sites did not improve significantly. In addition, although the thermostability score of the mutant E110Q was significantly improved, its activity score decreased more, and the composite score was only 4993.94, which was much lower than that of the wild type, so the composite mutation of these two sites was halted. For the single mutants of the other six sites, the results show that the composite score values of mutants N18L, S97A, G98R, S99L, G100A, and R143L were 14,570.10, 16,202.84, 19,892.83, 16,027.04, 19,429.07, and 20,604.41, respectively, representing the highest CSs among the mutants at their respective sites, with all being higher than the value of 10,000.00 of wild-type PB92. In summary, the composite mutation was continued for the six mutants N18L, S97A, G98R, S99L, G100A, and R143L. Considering that S97-S100 together constitute a loop, consider this region as a whole part and carry out composite mutations separately. Furthermore, compound mutations of N18L/R143L (M2), N18L/R143L/S97A (M3-1), N18L/R143L/G98R (M3-2), N18L/R143L/S99L, N18L/R143L/G100A (M3-4), and N18L/R143L/S97A/G98R/S99L/G100A (M6) were identified.

### 2.4. Characterization and Properties of Composite Mutants

**Figure 3:**
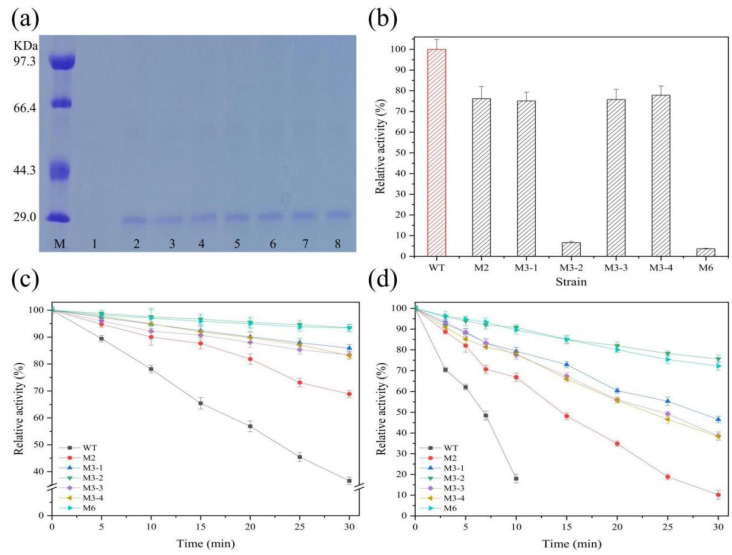
Expression and properties of complex mutants. (**a**) SDS–PAGE analysis of complex mutants. Lane M: protein molecular weight marker. Lane 1: the strain with an empty expression vector. Lanes 2–7: different mutants. Lane 8: wild-type protease PB92. (**b**) Activity comparison. (**c**) Thermostability comparison at 65 °C. (**d**) Thermostability comparison at 70 °C. WT represents an expression vector for the protease PB92, indicated by a red column or black lines. Mutants are indicated by a black column or lines of different colors. The high activity of wild-type protease PB92 was 3548.05 ± 80.39 U/mL at pH 10.5 and 55 °C, which was defined as 100%. Relative activity was defined as the percentage of measured high enzyme activity.

Six composite mutant plasmids were transformed into B. subtilis WB600 via electroporation. The sizes of the proteases were analyzed using sodium dodecyl sulfate-polyacrylamide gel electrophoresis (SDS-PAGE) analysis, and they were close to the predicted value (28.22 kDa) (Figure 3a). The comparison of activity between complex mutants and the wild type showed that the six complex mutants were reduced in activity, and the mutants M2, M3-1, M3-3, M3-4, and M3-5 retained more than 75%. However, the activities of mutants M3-2 and M7 decreased by more than 90% (Figure 3b). The residual activity between complex mutants and the wild type was compared at 65 °C and 70 °C, respectively (Figure 3c,d). At 65 °C, all six complex mutants exhibited good thermostability, and the residual activity remained above 75% after heat treatment for 30 min (Figure 3c). The residual activity remained above 25% when the six complex mutants were treated at 70 °C for 30 min, but that of the wild type was less than 20% after only 10 min (Figure 3d). The results showed that the thermostability of the complex mutants was greatly improved compared with that of the wild type.

Furthermore, the t_1/2_ values of different complex mutants at 65 °C were compared (Table 4). The t_1/2_ of wild-type protease was 23.14 min, and those of the complex mutants M2, M3-1, M3-2, M3-3, M3-4, and M6 were 49.00 min, 105.42 min, 232.45 min, 90.87 min, 91.42 min, and 224.62 min, respectively, which were 2.12–10.04 times higher than that of the wild type. In addition, although the thermostability of composite mutants M3-2 and M6 was the most obvious, their extracellular enzyme activity was also reduced by more than 90%, resulting in their composite score being lower than that of the wild type, which was not conducive to industrial production and application. The composite score values of the four composite mutants M2, M3-1, M3-3, and M3-4 were 16,141.99, 34,207.01, 29,739.52, and 30,752.94, respectively, which were higher than that of the wild type. In particular, the composite score values of M3-1, M3-3, and M3-4 were more than 3 times higher than that of the wild type, indicating good application prospects.

### 2.5. Structural Analysis of Improved Thermostability for Mutants

**Figure 4:**
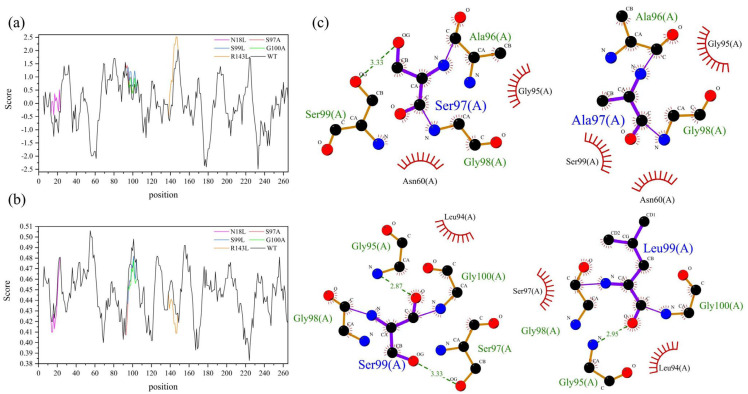
Structural analysis of complex mutants: (**a**) hydrophobicity of amino acid residues; (**b**) average flexibility of amino acid residues; (**c**) hydrogen bond and hydrophobic interaction analysis of wild-type and mutant proteins. Hydrophobicity and average flexibility analysis of amino acid residues were determined using the online software ProtScale (http://www.expasy.org/tools/protscale.html (accessed on 12 February 2023)).

To further develop the molecular mechanism of significantly improved thermostability for composite mutants M3-1, M3-3, and M3-4 containing five single mutation sites (N18L, S97A, S99L, G100A, and R143L), the hydrophobicity and flexibility of the wild type and five single mutant sites were investigated (Figure 4). The results showed that the scores of the five single mutation sites were higher than those of the wild type: the scores were increased from −0.722, 0.689, 0.178, 0.133, and 0.756 before the mutation to 0.089, 0.978, 0.689, 0.378, and 1.678 after the mutation, respectively (Figure 4a). The overall structure of the protein was also shown at the corresponding mutation point, with the color of the mutant tending to be brown, indicating higher hydrophobicity (Figure S4). The flexibility scores of the amino acid residues before the mutation of the N18, S97, S99, G100, and R143 sites were 0.432, 0.474, 0.490, 0.487, and 0.449, respectively, and the scores for the corresponding sites after mutation were 0.422, 0.458, 0.474, 0.467, and 0.431, respectively, indicating that the flexibility of the mutants was reduced (Figure 4b). To investigate the underlying structural differences contributing to distinct thermostabilities between the wild type and mutants, a comparative analysis was conducted between the crystal structure of the wild type and the structural models of the mutants. Notably, no significant alterations in the interactions were observed for N18L, G100A, and R143L in either the wild type or mutant structures (data not shown). However, the larger hydrophobic side chains in mutants S97A and S99L led to the formation of additional hydrophobic interactions (Ala97-Ser99 and Ser97-Leu99) that replaced the original hydrogen bonds (Figure 4c). Since thermostability is positively associated with hydrophobicity and negatively correlated with flexibility, the improved thermostability of composite mutants may be related to the formation of additional hydrophobic interactions due to increased hydrophobicity and the reduced flexibility of the structure.

**Figure 5:**
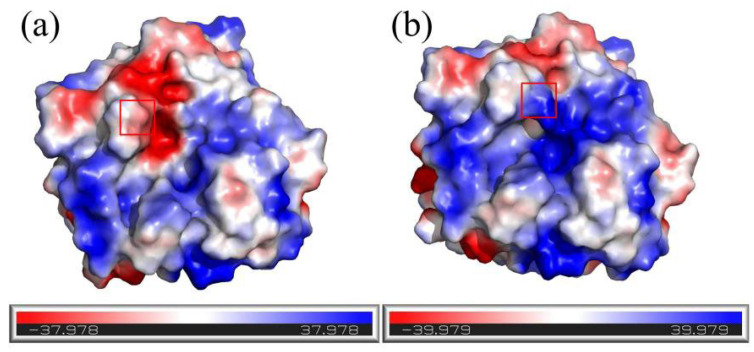
Surface electrostatic potential map of wild type and mutant G98R: (**a**) wild type; (**b**) mutant G98R. Positive, negative, and neutral values of electrostatic potentials are represented by shades of blue, red, and white, respectively.

Finally, the surface electrostatic potential of the wild type and mutant G98R was studied (Figure 5). The analysis of the electrostatic potential revealed that in the wild-type protease PB92 the amino acid residue G98 was situated within a negatively charged region on the protein surface (Figure 5a). However, upon mutating this site from Gly to Arg, arginine residues have larger side chain groups, forming a higher solvent accessible surface area (Figure S5). The resulting distribution of electrostatic potential deviated from the surrounding pattern, forming an electric double layer that shields the hydrophobic region on the protein surface. This alteration led to a positive shift in the potential within this region, making a significant positive contribution to the overall stability of the mutant (Figure 5b). Consequently, the favorable changes in the electrostatic potential on the protein surface, induced by the G98R mutation, facilitated the maintenance of a highly stable conformation to improve thermal stability.

## 3. Discussion

The development of thermostable proteases has significant potential to meet industrial demands. This is because such enzymes not only offer practical applications but also enhance kinetic efficiency [21,22]. In our study, the B-factor comparison, multiple sequence alignment, and composite score strategy were employed to guide the design and production of thermostable protease mutants. Through site-directed mutagenesis, three mutants were successfully produced: N18L/R143L/S97A, N18L/R143L/S99L, and N18L/R143L/G100A. These mutants exhibited higher thermostability and demonstrated considerable potential for industrial applications.

Firstly, with the rapid development of computational biology and structural biology, many parameters are provided for protein modification engineering, mainly including the temperature factor (B-factor), protein folding free energy, and other parameters to determine protein mutation sites and improve the interaction of related noncovalent forces to modify proteins, among which the B-factor is one of the most widely employed [22]. B-factor analysis of protease PB92 was the key step in the design of thermostable recombinant isoforms of this enzyme, given that B-factors reflect protein fluctuations and the rigidity of atoms in specific positions [25], and previous work has shown that a higher level of rigidity is known to improve thermostability [26]. As such, this study analyzed B-factor values to identify residues with pronounced degrees of flexibility for protease PB92 and aligned this sequence with those of other thermolysin sequences using the PDB, and then it subsequently engineered recombinant mutants by mutating these flexible residues. As a result, fifteen mutants exhibited increased t_1/2_ values (1.13–31.61 times greater than that of the WT) at 65 °C (Table 2). These findings confirm that B-factor analysis can effectively contribute to selectively enhancing the thermostability profiles of proteases.

Secondly, hydrophobic interactions play a key role in protein thermal stability by providing structural stability and folding energy. In general, the thermostability of enzymes is positively associated with their hydrophobicity and rigidity [27]. Research has indicated that thermophilic proteins contain a slightly higher number of hydrophobic amino acids compared to mesophilic proteins, as these residues enhance the hydrophobicity and thermostability of the protein [15]. It has also been found that hydrophobic interactions contribute approximately 64% to protein stability, while hydrogen bonding contributes 44% [28]. The results of this study showed hydrophobic amino acid substitutions resulted in a significant increase in hydrophobicity for loops 14–22, 96–101, and 139–147 in mutants (Figure 4a), and an analysis of the interactions near the corresponding wild-type and mutant sites demonstrated the elimination of hydrogen bonds and the creation of additional hydrophobic interactions between Ala97-Ser99 and Ser97-Leu99 (Figure 4c), contributing to the enhanced structural compactness and reduced perturbation after mutation, which could be one of the reasons for the improved thermostability of the mutants. Additionally, protein flexibility plays a crucial role in enzyme thermostability. Highly flexible residues can lead to protein unfolding, denaturation, and reduced enzyme activity at high temperatures [29]. The flexibility of our mutants N18L, S97A, S99L, G100A, and R143L was predicted such that the mutations resulted in a significant decrease in the flexibility of loops 14–22, 96–101, and 139–147 (Figure 4b), which may cause improved thermostability. Previous research has highlighted the presence of large hydrophobic surface areas and densely packed hydrophobic layers in thermophilic proteins. Therefore, the impact of introducing hydrophobic residues in flexible surface regions on protein thermal stability can be unexpected [30,31]. In our investigation, the additional hydrophobic interactions resulted in a more compact and rigid overall structure of the mutants, which likely contributed to a substantial enhancement in protease thermostability. It is well established that beneficial amino acid mutations often exhibit cumulative or synergistic effects on the thermal stability of proteins [32]. In alignment with this finding, our study demonstrated a synergistic effect on the thermostability of proteases by combining the advantageous amino acids present in mutations N18L/R143L/S97A, N18L/R143L/S99L, and N18L/R143L/G100A.

Thirdly, in our study, the thermostability of the complex mutants M3-2 and M6 increased nearly 10-fold, and it is not difficult to determine that this was caused by the mutant G98R. According to the literature, the thermostability of proteins is greatly affected by the surface charge, and the improvement of the thermostability of L-asparaginase, inulin sugar transferase, and agarose represents a method based upon the increase in surface potential [33,34,35], which is consistent with our results. This may be because the higher positive potential helps the enzyme protein to maintain a highly stable conformation, which improves the thermostability of the enzyme.

Fourthly, achieving both enhanced stability and activity in enzymes has long been recognized as a challenging endeavor due to the inherent trade-off between flexibility and rigidity within proteins [36]. The mechanism behind increased stability involves either an increase in rigidity or a decrease in flexibility, which can potentially have a detrimental effect on the catalytic activity of the enzyme [36,37]. Like L-asparaginase and β-glucanase, for which activity decreased with stabilization [33,37], in our study, the enzyme activity of the composite mutants (M3-1, M3-3, and M3-4) decreased over 30%, and even that of the complex mutants M3-5 and M6 decreased by more than 90% (Figure 3b). This may be potentially explained through the assertion that, because the mutation site is near the substrate binding pocket, the unfolding effect of the protease substrate binding site is weakened through direct interaction, and more substrate binding sites are embedded into the core of the protein, so that the exposure of the substrate channel is reduced, resulting in a significant decrease in enzyme activity. This is consistent with studies of β-glucanase [37]. However, in another study [38], the identification of a novel alkaline protease mutant G95P with enhanced specific activity, thermostability, and alkaline stability, especially a 9-fold increase in specific activity, from a library of mutants constructed using error-prone PCR, was superior to our study. From the thermal stability data, it is not as good as our study; whether the advantages of the two studies can be superimposed is worthy of further study. In addition, the importance of the loop region was also mentioned in that study, which coincides with our study. The effect of S97–S101 on the properties of the PB92 may only be further revealed by the strategy of error-prone PCR.

## 4. Materials and Methods

### 4.1. Strains and Chemicals

High-fidelity DNA polymerase, restriction endonucleases (BamHI, SalI), and dNTPs were purchased from TaKaRa (Kunming, China). The plasmid mini-prep kit and DNA gel extraction kit were procured from Omega (Kunming, China). A one-step cloning kit was purchased from Vazyme Biotech (Nanjing, China). The Fast MultiSite Mutagenesis System, *Escherichia coli* Trans I-T1, and DMT cells were obtained from TransGen (Beijing, China). All other chemicals were of an analytical grade and commercially available.

### 4.2. Selection of the Target Residues for Improving the Thermostability of Protease PB92

Thermolysin sequences were searched in the Protein Data Bank (https://www.rcsb.org/ (accessed on 24 October 2022)) to perform multiple sequence alignments of sequences derived from Bacillus with the protease PB92. For the site of B-factor > 21.0 of the C_α_ atom of the amino acid residue in the crystal structure of protease PB92, the statistics of multisequence alignment results were calculated, and the top two or more than 10% amino acid residues with the conservation of each site were confirmed as mutant amino acids.

### 4.3. Site-Directed Mutagenesis Strategy

The complete open-reading frame of protease PB92 was 1143 bp (NCBI accession number: WP_094423791.1), and recombinant plasmid pBE-09-PB92 as a mutation template was constructed using primers PB92-F and PB92-R (Table S2), containing a strong promoter, pShuttle-09 [24]. The primers required for the mutagenesis were synthesized according to Table S2. The mutations were introduced into the plasmid using the whole plasmid PCR method [22]. In a 20 μL reaction system, the template (pBE-09-PB92) was added at a concentration of 2 ng, along with 1 μL each of the upstream and downstream primers (10 μM). The PCR SuperMix (2×) and ddH_2_O completed the reaction mixture. The PCR cycling protocol consisted of an initial denaturation step at 95 °C for 5 min, followed by denaturation at 94 °C for 30 s, annealing at 53 °C for 30 s, and extension at 72 °C for 5 min, repeated for 30 cycles, with a final complete extension at 72 °C for 10 min. The resulting PCR products were subjected to digestion with 0.6 μL of DMT enzyme for 3 h on methylated or hemimethylated parental templates. Subsequently, the digested products were transformed into *E. coli* DMT, and sequencing analysis was performed to confirm the accuracy of the mutant sequences.

### 4.4. Expression, Purification, and Activity Assay of Protease PB92

The recombinant plasmids containing the wild-type and mutant sequences were successfully transformed into *B. subtilis* WB600 [39]. A single colony that displayed a transparent circle on a plate containing 50 mg/mL kanamycin and 1% skim milk powder was screened. This colony was then inoculated in 5 mL of LB broth and incubated at 37 °C to establish a seed culture. Subsequently, a fresh superrich medium composed of 10 g/L glucose, 25 g/L yeast extract, 3 g/L K_2_HPO_4_, 25 g/L tryptone, and 10 g/L NaCl was prepared and inoculated with 5% of the seed culture in a 500 mL flask. The culture was further incubated at 37 °C for 60 h. After incubation, the medium was subjected to centrifugation at 12,000× *g* for 20 min to obtain a crude enzyme solution.

The crude enzyme solution was concentrated 20-fold using an ultrafiltration centrifugal tube (Solarbio, Beijing, China). Subsequently, the recombinant protease, which was tagged with His at the C-terminus, underwent purification using an agarose gel column. Elution was carried out employing an ice-cold buffer (pH 7.5) consisting of 15 mmol/L Tris-HCl and 0.5 mol/L NaCl, with a continuous gradient of imidazole concentrations ranging from 0 to 200 mmol/L. The purity of the protein was assessed using SDS-PAGE.

Protease activity was determined using azocasein (Sigma, Shanghai, China) as a substrate with slight modifications based on the methodology described by Cabral et al. [24,40]. One unit of enzyme activity was defined as the amount of enzyme that hydrolyzes 1 μg of azocasein per min under standard assay conditions. The reaction system consisted of 450 μL of a glycine–NaOH buffer (100 mM, pH 11.0), 450 μL of the substrate stock solution (1.0% *w*/*v*) of azocasein, and 100 μL of a diluted enzyme solution. Following incubation for 10 min at 40 °C, the reaction was terminated by adding 1.0 mL of trichloroacetic acid (10.0% *w*/*v*), and the sample was incubated in an ice bath for 10 min. Subsequently, the solution was subjected to centrifugation, and the absorbance of the supernatant was measured at 405 nm using a SpectraMax instrument. A negative control without the enzyme was included in each experiment. Triplicate measurements were performed for all samples to ensure accuracy and reliability.

### 4.5. Enzymatic Characterization of Protease PB92

The optimal temperature for protease PB92 activity was determined by screening at 40–75 °C at pH 11.0. To assess thermostability, the enzyme was preincubated for different times at 65–70 °C, followed by a measurement of residual activity at pH 10.5 and 55 °C. The resulting data were fitted to a curve, and the time required for the enzyme activity to decrease by half was defined as the half-life (t_1/2_).

### 4.6. Identification and Implementation of Complex Mutants

A composite score was used to identify complex mutants, with some modifications as described in Li et al. [41]. The relative enzymatic activity and thermostability of single-mutants and wild-type proteases were scored, respectively. The activity of mutants relative to 1% changes in the wild type was defined as a score of 1, and the activity score of the wild type was defined as 100. The t_1/2_ of mutants relative to 1% changes in the wild type was defined as a score of 1, and the thermostability score of the wild type was defined as 100. The composite score was defined as the weighting of the thermostability score and activity score. In this study, the importance of thermostability and activity for the proteinase PB92 was equal, and the weight coefficient was 1. The single mutation with the highest composite score per locus was used to perform complex mutations. A positive mutant displaying significantly improved thermostability was selected as the template. Using the aforementioned method, amino acid mutations were introduced at other locations in the protein sequence [22].

### 4.7. Molecular Modeling and Structural Analysis

The crystal structure of protease PB92 isolated from *B. alcalophilus* (PDB ID: 1IAV) [5] served as the reference template for constructing the three-dimensional (3D) structures of the mutants using the Swiss-Model online platform (https://swissmodel.expasy.org/ (accessed on 24 October 2022)). The resulting models were subjected to comparative analysis using PyMOL visualization software (Schrodinger, LLC 4.60), while the interaction between mutant amino acids and other residues was examined using Discovery Studio Visualizer software 2021. The hydrophobicity and flexibility scores for each amino acid residue were calculated using ProtScale, an online software tool (http://www.expasy.org/tools/protscale.html (accessed on 12 February 2023)). The solvent-accessible surface area of mutant G98R was calculated using gmx hbond [42]. The surface charged amino acids of the wild type and mutants were displayed using WebLogo, and the surface potential and structure visualization were presented using PyMOL.

## 5. Conclusions

Overall, protease PB92 with high thermostability was generated by a rational approach. To the best of our knowledge, this is the first study to design protease PB92 with improved thermostability via composite scores combined with B-factor comparison and multiple sequence alignment. Furthermore, 3D structure analysis revealed that increased hydrophobicity, decreased flexibility, and hydrophobic interactions were introduced for mutants N18L/R143L/S97A, N18L/R143L/S99L, and N18L/R143L/G100A with improved thermostability. This study provides valuable insights and a rational and efficient approach for enhancing the thermostability of proteases, thus facilitating their application in industrial processes operating at high temperatures.

## Figures and Tables

**Table 1:** Comparison of activities and Topt between mutants and wild type ^a^.

Protease	Relative Activity (%) ^b^	Topt (°C) ^c^	Protease	Relative Activity (%) ^b^	Topt (°C) ^c^
Wild type ^d^	100	55	G100E	7.88	60
N18L	72.30	60	G100A	93.82	55
N18Q	0.23	ND	S101I	3.26	ND
S97A	90.89	55	S101G	90.29	55
S97H	98.52	55	S101V	12.69	65
G98R	19.95	55	E110L	31.28	60
G98E	6.19	55	E110R	0.12	ND
G98Q	3.45	ND	R143F	0.02	ND
S99L	96.66	60	R143L	75.73	60
S99A	111.01	60	R143G	98.24	60
G100K	0.15	ND	R143V	78.53	55

^a^ ND: not detected. ^b^ Relative activity: the enzymatic activity of protease PB92 was found to be significantly high at pH 10.5 and 55 °C. The wild-type protease PB92 exhibited an enzyme activity of 3548.05 ± 80.39 U/mL, which was designated as the reference value of 100%. Relative activity denotes the percentage of measured enzymatic activity compared to the reference value of the wild type, thus reflecting the relative efficiency of the tested variants. ^c^ Topt: the enzyme activity was determined at different temperatures of 40–75 °C and pH 10.5. ^d^ Wild type: protease PB92.

**Table 2:** The half-life (t_1/2_) values of mutants and wild type at 65 °C.

Protease	t_1/2_ (min) ^a^	Increase Times	Protease	t_1/2_ (min) ^a^	Increase Times
Wild type ^b^	23.14	1.00	G100E	548.80	23.72
N18L	46.63	2.02	G100A	47.92	2.07
S97A	41.25	1.78	S101G	28.18	1.22
S97H	26.16	1.13	S101V	190.44	8.23
G98R	230.71	9.97	E110L	36.94	1.60
G98E	731.39	31.61	R143V	49.81	2.15
S99L	38.37	1.66	R143L	62.96	2.72
S99A	32.40	1.40	R143G	28.14	1.65

^a^ t_1/2_: After the enzyme was preincubated at 65 °C for different times, thermostability was determined at pH 10.5 and 55 °C. The resulting data points of residual activity versus time were fitted to a curve to determine the half-life (t_1/2_) of the enzyme. The t_1/2_ represents the time required for the enzyme’s activity to decrease by half under the given experimental conditions. ^b^ Wild type: protease PB92.

**Table 3:** Activity score, thermostability score, and composite score between mutants and wild type.

Protease	Thermostability Score ^a^	Activity Score ^b^	Composite Score ^c^	Protease	Thermostability Score ^a^	Activity Score ^b^	Composite Score ^c^
Wild type ^d^	100	100	10,000.00	G100E	237.17	7.88	18,691.55
N18L	201.51	72.3	14,570.10	G100A	207.09	93.82	19,429.07
S97A	178.26	90.89	16,202.84	S101G	121.79	94.23	10,996.84
S97H	113.47	98.52	11,178.42	S101V	822.99	12.69	10,445.30
G98R	997.02	19.95	19,892.83	E110L	159.64	31.28	4993.94
G98E	316.07	6.19	19,552.51	R143V	215.25	78.53	16,903.36
S99L	165.82	96.66	16,027.04	R143L	272.07	75.73	20,604.41
S99A	140.02	111.01	15,543.38	R143G	164.83	98.24	16,192.72

^a^ Thermostability score: the t_1/2_ of mutants relative to 1% changes in the wild type was defined as a score of 1, and the wild type was defined as 100. ^b^ Activity score: the activity of mutants relative to 1% changes in the wild type was defined as a score of 1, and the wild type was defined as 100. ^c^ The composite score was defined as the weighting of the thermostability score and activity score. In this study, the importance of thermostability and activity for the protease PB92 was equal, and the weight coefficient was 1. ^d^ Wild type: protease PB92.

**Table 4:** The half-life (t_1/2_) and composite score of complex mutants and wild type.

Protease	t_1/2_ (min) ^a^	Increase Times	Composite Score ^b^
Wild type ^c^	23.14	1.00	10,000
M2	49.00	2.12	16,141.99
M3-1	105.42	4.55	34,207.01
M3-2	232.45	10.04	6692.22
M3-3	90.87	3.93	29,739.52
M3-4	91.42	3.95	30,752.94
M6	224.62	9.71	3544.60

^a^ t_1/2_: the t_1/2_ represents the time required for the enzyme’s activity to decrease by half under the given experimental conditions. ^b^ The composite score was defined as the weighting of the thermostability score and activity score. In this study, the importance of thermostability and activity for the protease PB92 was equal, and the weight coefficient was 1. ^c^ Wild type: protease PB92.

## Data Availability

The data used to support the findings of this study can be made available by the corresponding author upon request.

## Supplementary Materials

The following supporting information can be downloaded at: https://www.mdpi.com/article/10.3390/foods12163081/s1, Figure S1: The transparent circle formed for different mutants, Figure S2: Comparison of enzyme activity of different mutants, Figure S3: The optimal temperature for different mutants of protease PB92, Figure S4: Overall hydrophobicity of the protein, Figure S5: The solvent-accessible surface area of mutant G98R, Table S1: The frequencies of 20 different amino acids were counted that the 8 amino acid sites corresponding to protease PB92 via multi-sequence alignment, Table S2: Primers used in the present research.
