# Supplementary material for: Improving the Thermostability of Serine Protease PB92 from Bacillus alcalophilus via Site-Directed Mutagenesis Based on Semi-Rational Design
Source: Foods. 2023 Aug 16;12(16):3081. doi: 10.3390/foods12163081 (PMC10453622; doi:10.3390/foods12163081)
Supplement: Supplementary file 1 [file foods-12-03081-s001.zip › foods-2541907-supplementary.pdf]

Supporting Information

Improving the Thermostability of Serine Protease PB92 from *Bacillus alcalophilus* via Site-Directed Mutagenesis Based on Semi-Rational Design

Huabiao Miao [1,2], Xia Xiang [2], Nanyu Han [1,2], Qian Wu [1,2] and Zunxi Huang [1,2,*]

1. Engineering Research Center of Sustainable Development and Utilization of Biomass Energy, Ministry of Education, Kunming 650500, China
2. School of Life Science, Yunnan Normal University, Kunming 650500, China

* Correspondence: huangzunxi@ynnu.edu.cn; Tel.: +86-0871-5920830; Fax: +86-0871-5920952

The transparent circle formed for different mutants. CK represents the control group, the strain with an empty expression vector. WT represents the wild type group, the strain with an expression vector of protease PB92. Others represents the mutant group, the strain with different mutant plasmids of protease PB92. All single colonies are inoculated overnight on plates containing 50 mg/mL kanamycin and 1% skim milk powder at 37°C.

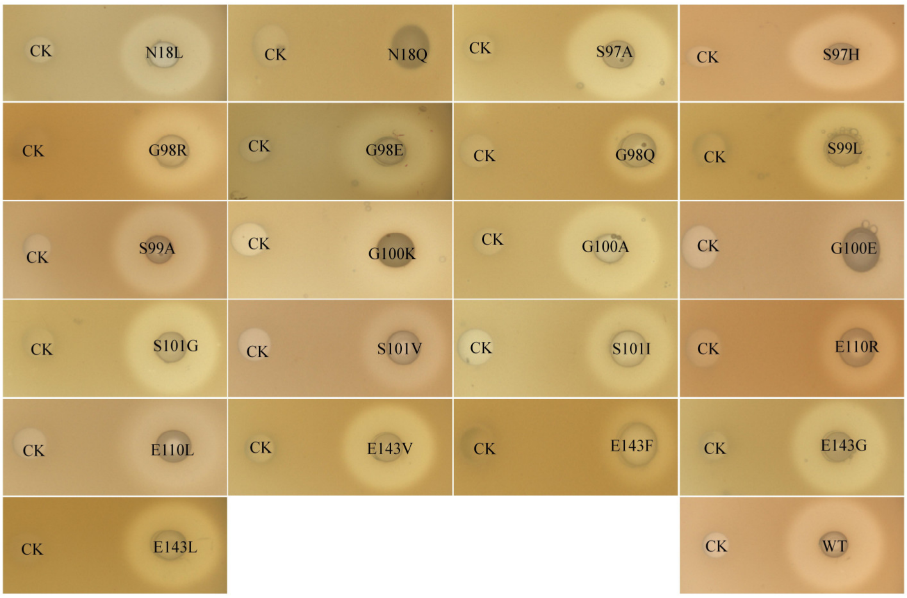

Figure S1. The transparent circle formed for different mutants.

Comparison of enzyme activity of different mutants. (a) Wild type, and mutants of N18 and S97. (b) Mutants of G98 and S99. (c) Mutants of G100 and S101. (d) Mutants of E110 and R143. WT represents the wild type group, which has an expression vector for the protease PB92, indicated by a red column. Others represents the mutant group, the strain with different mutant plasmids of protease PB92, indicated by a black column. The enzyme activity demonstrated high activity at pH 10.5 and 55°C. The enzyme activity of wild-type protease PB92 was 3548.05 ± 80.39 U/mL , which was defined as 100%. Relative activity was defined as the percentage of measured high enzyme activity. All values are presented as the mean ± SD of triplicate experiments.

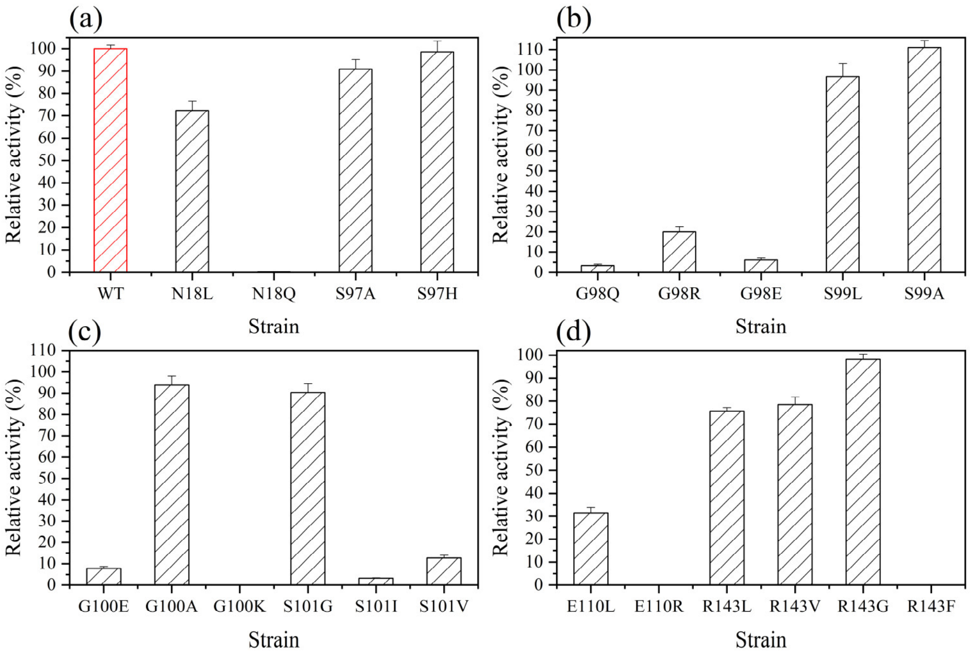

Figure S2. Comparison of enzyme activity of different mutants.

The optimal temperature for different mutants of protease PB92. (a) Wild type, and mutants N18L, S99A, and S99L. (b) Mutants S97H, S97A, G98E, and G98R. (c) Mutants G100E, G100A, S101G, and S101V. (d) Mutants E110L, R143V, R143L, and R143G. WT represents the wild type group, which has an expression vector for the protease PB92. Others represents the mutant group, the strain with different mutant plasmids of protease PB92. The enzyme activity was determined at different temperatures of 40-75°C and pH 10.5. The enzyme activity demonstrated high activity, which was defined as 100%. Relative activity was defined as the percentage of measured high enzyme activity. All values are presented as the mean ± SD of triplicate experiments.

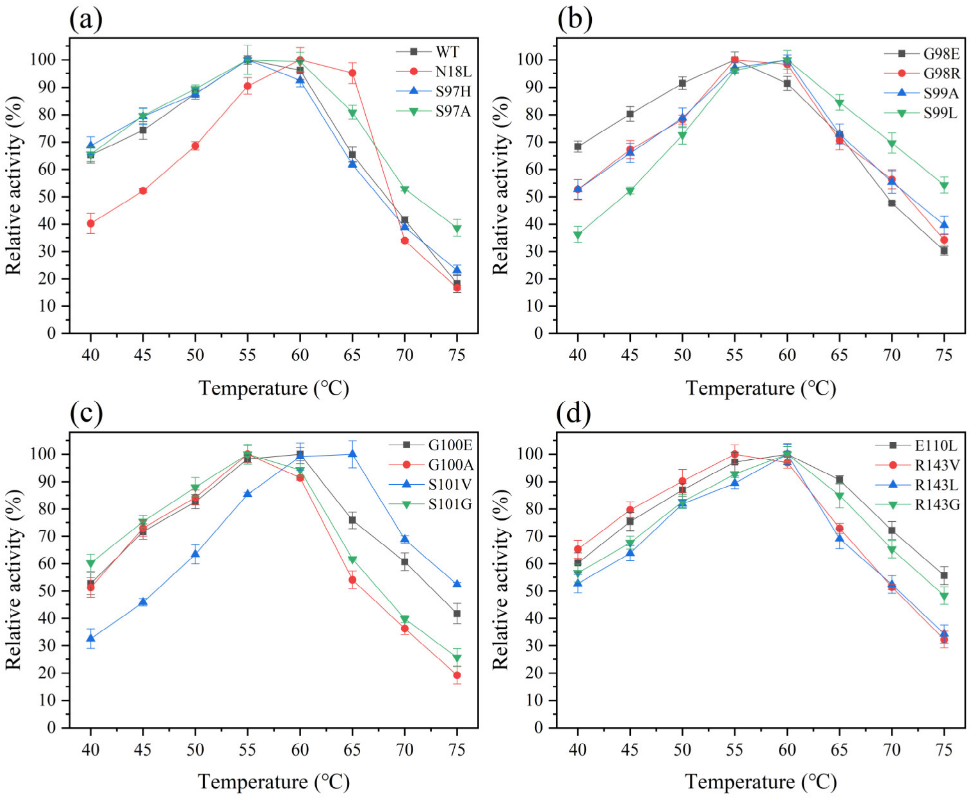

Figure S3. The optimal temperature for different mutants of protease PB92.

Overall hydrophobicity of the protein. Overall hydrophobicity of protein and interaction between mutant amino acids with other residues determined using the software Discovery Studio Visualizer.

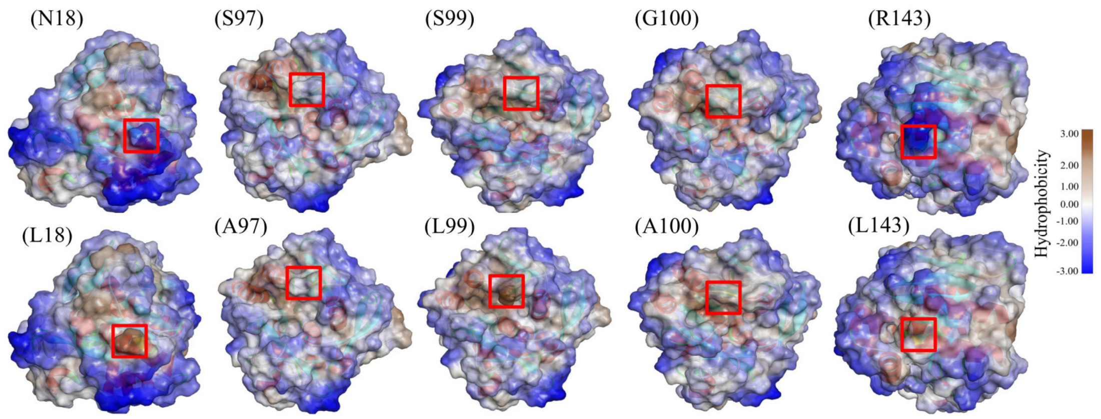

Figure S4. Overall hydrophobicity of the protein.

The solvent-accessible surface area of mutant G98R. (a) Gly98. (b) Arg98. (c) Comparison of solvent-accessible surface area. The solvent-accessible surface area of mutant G98R was calculated using gmx hbond, and the sasa_dots and structure visualization were presented using PyMOL.

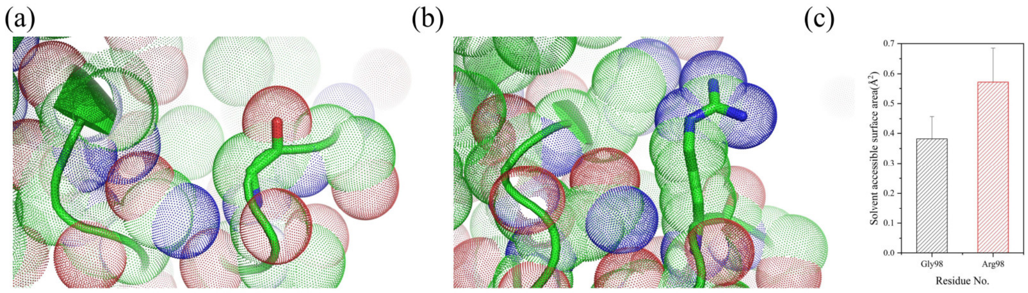

Figure S5. The solvent-accessible surface area of mutant G98R.

Table S1. The frequencies of 20 different amino acids were counted that the 8 amino acid sites corresponding to protease PB92 via multi-sequence alignment.

%

Item	N18	S97	G98	S99	G100	S101	E110	R143
D	7.83	6.59	6.98	2.06	8.25	3.88	5.00	5.56
K	4.35	4.40	5.81	6.19	11.34	6.80	6.25	5.56
H	1.74	9.89	1.16	1.03	3.09	3.88	2.50	1.85
L	13.04	7.69	5.81	16.49	5.15	3.88	21.25	9.26
E	5.22	3.30	12.79	5.15	13.40	1.94	0.00	4.63
Y	4.35	7.69	3.49	4.12	4.12	0.00	3.75	4.63
A	6.96	16.48	5.81	12.37	14.43	7.77	5.00	6.48
S	4.35	0.00	8.14	0.00	2.06	0.00	3.75	2.78
I	6.09	5.49	1.16	8.25	6.19	12.62	6.25	6.48
Q	8.70	1.10	10.47	2.06	1.03	6.80	3.75	6.48
C	0.87	2.20	1.16	0.00	0.00	0.97	0.00	0.00
F	4.35	3.30	3.49	1.03	7.22	1.94	3.75	9.26
W	5.22	2.20	0.00	0.00	2.06	0.97	0.00	1.85
M	6.09	3.30	0.00	1.03	2.06	3.88	1.25	3.70
R	6.09	3.30	13.95	7.22	5.15	3.88	12.50	0.00
G	6.09	6.59	0.00	9.28	0.00	14.56	3.75	10.19
V	4.35	5.49	2.33	7.22	4.12	12.62	6.25	9.26
T	3.48	5.49	2.33	7.22	3.09	1.94	5.00	2.78

P	0.87	3.30	9.30	7.22	6.19	7.77	3.75	2.78
N	0.00	2.20	5.81	2.06	1.03	3.88	6.25	6.48
Total	100	100	100	100	100	100	100	100

Table S2. Primers used in the present research.

Primer	Primer Sequence (5'-3')
PB92-F	TGCGGCGTCCATAAAGTGGGATCCATGAAGAAACCGTTGGGGAAAATTG
PB92-R	GTGATGTCTAGACTGCAGGTCGACGCGTGTTGCCGCTTCTGCATTGACA
N18L-F	AAGCCCCAGCTGCCCAT**TTA**CGTGGATTGACA
N18L-R	**TAA**ATGGGCAGCTGGGGCTTGCACACGGCTAA
N18Q-F	AAGCCCCAGCTGCCCAT**CAA**CGTGGATTGACA
N18Q-R	**TTG**ATGGGCAGCTGGGGCTTGCACACGGCTAA
S97A-F	TTAAAGTATTAGGGGCG**GCC**GGTTCAGGTT
S97A-R	**GGC**CGCCCCTAATACTTTAACAGCGTATAGTT
S97H-F	TTAAAGTATTAGGGGCG**CAC**GGTTCAGGTT
S97H-R	**GTG**CGCCCCTAATACTTTAACAGCGTATAGTT
G98R-F	AAGTATTAGGGGCGAGC**CGT**TCAGGTTCGGT
G98R-R	**ACG**GCTCGCCCCTAATACTTTAACAGCGTATA
G98Q-F	AAGTATTAGGGGCGAGC**CAG**TCAGGTTCGGT
G98Q-R	**CTG**GCTCGCCCCTAATACTTTAACAGCGTATA
G98E-F	AAGTATTAGGGGCGAGC**GAA**TCAGGTTCGGT
G98E-R	**TTC**GCTCGCCCCTAATACTTTAACAGCGTATA
S99L-F	TATTAGGGGCGAGCGGT**TTA**GGTTCGGTCAGC
S99L-R	**TAA**ACCGCTCGCCCCTAATACTTTAACAGCGTAT
S99A-F	TATTAGGGGCGAGCGGT**GCA**GGTTCGGTCAGC
S99A-R	**TGC**ACCGCTCGCCCCTAATACTTTAACAGCGTAT
G100E-F	TAGGGGCGAGCGGTTCA**GAA**TCGGTCAGCTCG
G100E-R	**TTC**TGAACCGCTCGCCCCTAATACTTTAACAGCGT
G100A-F	TAGGGGCGAGCGGTTCA**GCT**TCGGTCAGCTCG
G100A-R	**AGC**TGAACCGCTCGCCCCTAATACTTTAACAGCGT

G100K-F	TAGGGGCGAGCGGTTCA**AAG**TCGGTCAGCTCG
G100K-R	**CTT**TGAACCGCTCGCCCCTAATACTTTAACAGCGT
S101G-F	GGGCGAGCGGTTCAGGT**GGA**GTCAGCTCGATT
S101G-R	**TCC**ACCTGAACCGCTCGCCCCTAATACTTTAA
S101I-F	GGGCGAGCGGTTCAGGT**ATA**GTCAGCTCGATT
S101I-R	**TAT**ACCTGAACCGCTCGCCCCTAATACTTTAA
S101V-F	GGGCGAGCGGTTCAGGT**GTA**GTCAGCTCGATT
S101V-R	**TAC**ACCTGAACCGCTCGCCCCTAATACTTTAA
E110L-F	CGATTGCCCAAGGATTG**CTA**TGGGCAGGGAAC
E110L-R	**TAG**CAATCCTTGGGCAATCGAGCTGACCGAAC
E110R-F	CGATTGCCCAAGGATTG**CGA**TGGGCAGGGAAC
E110R-R	**TCG**CAATCCTTGGGCAATCGAGCTGACCGAAC
R143L-F	TTAATAGCGCGACTTCT**TTA**GGCGTTCTTGTT
R143L-R	**TAA**AGAAGTCGCGCTATTAACAGCTTGCTC
R143V-F	TTAATAGCGCGACTTCT**GTA**GGCGTTCTTGTT
R143V-R	**TAC**AGAAGTCGCGCTATTAACAGCTTGCTC
R143G-F	TTAATAGCGCGACTTCT**GGA**GGCGTTCTTGTT
R143G-R	**TGG**AGAAGTCGCGCTATTAACAGCTTGCTC
R143F-F	TTAATAGCGCGACTTCT**TTC**GGCGTTCTTGTT
R143F-R	**GAA**AGAAGTCGCGCTATTAACAGCTTGCTC

The *BamH*I and *Sal*I site sequences are underlined, and the mutation site is bolded.
